# Suppression of Endogenous Glucose Production by Isoleucine and Valine and Impact of Diet Composition

**DOI:** 10.3390/nu8020079

**Published:** 2016-02-15

**Authors:** Isabel Arrieta-Cruz, Ya Su, Roger Gutiérrez-Juárez

**Affiliations:** 1 National Institute of Geriatrics, Ministry of Health, Periférico Sur No. 2767, Col. San Jerónimo Lídice. Del. Magdalena Contreras, Mexico City 10200, Mexico; arrieta777@mail.com; 2 Department of Medicine, Albert Einstein College of Medicine, Yeshiva University, 1300 Morris Park Ave, Bronx, NY 10461, USA; ya.su@einstein.yu.edu

**Keywords:** branched-chain amino acids, leucine, isoleucine, valine, glucose metabolism, nutrient sensing, hypothalamus, liver

## Abstract

Leucine has been shown to acutely inhibit hepatic glucose production in rodents by a mechanism requiring its metabolism to acetyl-CoA in the mediobasal hypothalamus (MBH). In the early stages, all branched-chain amino acids (BCAA) are metabolized by a shared set of enzymes to produce a ketoacid, which is later metabolized to acetyl-CoA. Consequently, isoleucine and valine may also modulate glucose metabolism. To examine this possibility we performed intrahypothalamic infusions of isoleucine or valine in rats and assessed whole body glucose kinetics under basal conditions and during euglycemic pancreatic clamps. Furthermore, because high fat diet (HFD) consumption is known to interfere with central glucoregulation, we also asked whether the action of BCAAs was affected by HFD. We fed rats a lard-rich diet for a short interval and examined their response to central leucine. The results showed that both isoleucine and valine individually lowered blood glucose by decreasing liver glucose production. Furthermore, the action of the BCAA leucine was markedly attenuated by HFD feeding. We conclude that all three BCAAs centrally modulate glucose metabolism in the liver and that their action is disrupted by HFD-induced insulin resistance.

## 1. Introduction

Excessive calorie intake resulting from the consumption of fat-rich diets is the most important environmental factor contributing to the emergence and worsening of the current world pandemic of diabetes and obesity [1,2]. Mammals have developed complex mechanisms for detecting changes in the availability of nutrients and responding to them with metabolic adaptations to maintain homeostasis. Multiple organs and tissues are involved in these physiological adaptive mechanisms of nutrient sensing, but the mediobasal hypothalamus (MBH) of the central nervous system (CNS) has been identified as a key integration center for these nutritional cues [3,4,5,6]. Dietary protein and amino acids (AA) exert a powerful influence on insulin action and glucose metabolism. The mechanisms underlying this effect are generally attributed to the metabolic actions of AAs in the liver and skeletal muscle [7,8,9,10,11,12,13]. Recently however, a number of metabolic actions of AAs have been localized to the MBH of rodents [14,15,16,17]. In particular, we have shown that the metabolism of leucine to acetyl-CoA in the MBH is coupled to the inhibition of endogenous glucose production (EGP) and a consequent decrease of circulating glucose levels [16]. In the brain, leucine is initially metabolized through the consecutive action of five enzymes [18,19]. First, leucine is transaminated to *α*-KIC by the branched-chain amino transferase (BCAT). Next, *α*-KIC is oxidatively decarboxylated by the action of the branched-chain *α*-ketoacid dehyrogenase (BCKDH) to form isovaleryl-CoA, which, after three more reactions, is converted to acetyl-CoA. In our studies [16], interventions that antagonized the activity of the enzyme BCKDH or otherwise prevented the formation of acetyl-CoA in the MBH markedly attenuated the glucoregulatory action of leucine. Thus, the conversion of leucine to acetyl-CoA was required to bring about the glucoregulatory effect. Further experiments also revealed that acetyl-CoA had to be converted to malonyl-CoA. More importantly, we showed that the incapacitation of leucine sensing in the MBH contributes to the development of hyperglycemia [16]. Interestingly, the first two enzymes, BCAT and BCKDH, also catalyze the metabolism of the other two members of the branched-chain amino acids (BCAA) family, isoleucine and valine, to form their respective ketoacid products. Successive enzymatic reactions give rise to various metabolites, including acetyl-CoA, a key intermediate for glucoregulation.

Several studies have shown that acute diet-induced insulin resistance partially obliterates the hypothalamic glucoregulatory response to fatty acids [20,21,22]. This acquired defect is induced by the consumption of a diet enriched in saturated fat. Furthermore, previous studies indicated that the hypothalamic sensing of glucose and lactate, both of which need to be converted to pyruvate, are attenuated by high-fat diet (HFD) feeding [23]. Importantly, studies in rats revealed that HFD feeding caused a marked decrease in the levels of hypothalamic long-chain fatty acyl CoAs (LCFA-CoA) and that their restoration normalized nutrient-dependent glucoregulation [22]. Based on these findings, we hypothesized that HFD feeding may also perturb the glucoregulatory response to BCAA.

Currently it is not known whether or not isoleucine and valine modulate glucose metabolism; furthermore, the effect of HFD on the central metabolic actions of BCAA has not yet been examined. Here we investigated the possibility that the central metabolism of isoleucine and valine may also be coupled to the regulation of glucose metabolism in the liver. Additionally, to determine whether HFD feeding has an impact on the glucoregulatory action of BCAAs, we infused leucine in the MBH of rats previously fed with a lard-enriched diet. In both cases we assessed glucose metabolism *in vivo* through a combination of pancreatic insulin clamps and measurements of whole body glucose kinetics.

## 2. Experimental Section

### 2.1. Animal Studies

The animal studies were approved by the Institutional Animal Care and Use Committee (IACUC) of the Albert Einstein College of Medicine. Ten-week-old Sprague-Dawley male rats (Charles River Laboratories, Wilmington, MA, USA) were used for the studies. The animals were individually housed and subjected to a light–dark cycle (0600–1800/1800–0600) with free access to water and food.

#### 2.1.1. Animal Surgeries

Rats were subjected to stereotaxic surgery for implantation of a stainless steel bilateral cannula in the MBH as previously described [16]. The stereotaxic coordinates for cannula placement were (from bregma): −3.3 mm anterior–posterior axis; 0.4 mm lateral axis; and 9.6 mm vertical axis (depth) [24]. On recovery, the animals underwent a second surgery for the placement of indwelling vascular catheters that were used for infusions and blood sampling during the pancreatic clamp studies [16]. Postoperative recovery was monitored by measuring daily food intake and body weight. Only fully recovered animals were used for the experiments. Proper placement of the cannulas was confirmed histologically in brain slices prepared postmortem.

#### 2.1.2. Diets

In the experiments where we compared leucine, isoleucine, and valine, the rats were fed with a regular chow diet (Cat#5001, Lab Diet, Richmond, IN, USA). In separate experiments designed to test the effect of saturated fat on leucine sensing, the animals were subjected to a 3-day regime of a HFD consisting of regular chow enriched with 10% of lard (Cat#01P5704C-K, Test Diet, Richmond, IN, USA) prior to the clamps.

#### 2.1.3. Intrahypothalamic Infusions

Rats were randomized into three groups and received 6 hr intrahypothalamic (MBH) infusions of the following solutions: (1) Vehicle (artificial cerebrospinal fluid); (2) isoleucine; and (3) valine. For the glucose kinetics experiments, an additional group of animals received leucine at an equimolar dose for comparative purposes. Each AA was dissolved in vehicle and delivered at a total dose of 12 *n*moles over the course of 6 h (0.33 μL/h per side). The protocol and dose was based on our previous *in vivo* studies with leucine [16]. A separate group of rats were subjected to HFD feeding and then similarly infused with leucine during pancreatic clamps.

#### 2.1.4. Pancreatic Clamp Studies and Glucose Kinetics Measurements

**Figure 1:**
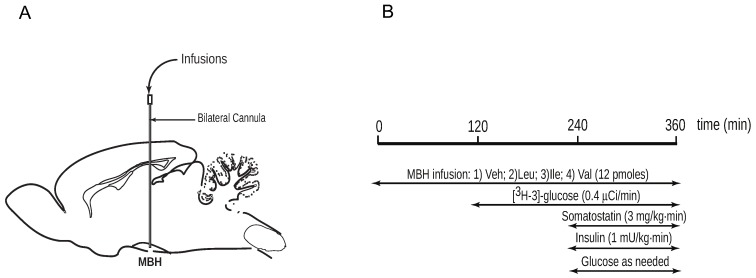
Experimental procedures. (**A**) Schematic of rat brain (sagittal view) with bilateral cannula implanted stereotactically for infusions into the mediobasal hypothalamus (MBH); (**B**) Time-line for *in vivo* central infusions of branched-chain amino acids (BCAAs) during euglycemic pancreatic clamp protocol with isotopic glucose tracer.

All rats on regular chow consumed 60 kcal of food the night prior to the study to ensure comparable nutritional status. Plasma levels of circulating glucose were monitored from the start (t = 0 min) of the central infusion (Figure 1A,B). A primed, continuous infusion of [3-${}^{3}$H] glucose (Perkin Elmer, San Jose, CA, USA; 40 μCi bolus; 0.4 μCi/min) was initiated at t = 120 min and maintained throughout the study to assess glucose kinetics by tracer dilution methodology [25], then a pancreatic clamp with insulin replaced at basal levels was performed (t = 240–360 min). At the end of the study, the animals were euthanized and tissue samples were freeze-clamped *in situ* and stored for subsequent analysis.

### 2.2. Analytical Procedures and Calculations

Plasma glucose was measured using an Analox instrument (Analox Instruments USA Inc., Lunenburg, MA, USA). The radioactivity of [3-${}^{3}$H] glucose in plasma was measured from supernatants of Ba(OH)${}_{2}$ and from ZnSO${}_{4}$ precipitates (Somogyi procedure) of plasma samples after each was evaporated to dryness to remove tritiated water. The rate of glucose uptake and endogenous glucose production were calculated as previously described [26].

### 2.3. Statistical Analysis

All data values are expressed as mean ± S.E.M. of the indicated number of experiments. Statistical comparisons were assessed by unpaired Student’s *t* test or analysis of variance (ANOVA) followed by the Tukey HSD test. We used the customary threshold of *p* < 0.05 to declare statistical significance.

## 3. Results

**Figure 2:**
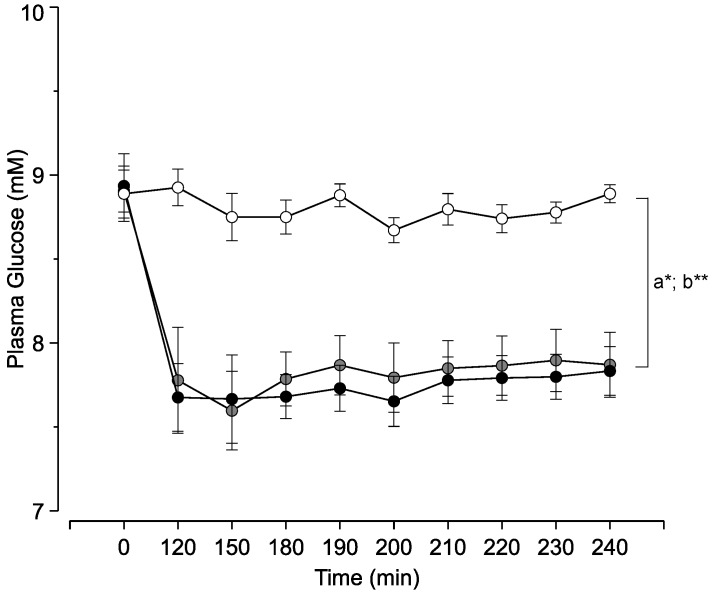
Effect of central isoleucine and valine on circulating glucose levels. Symbols (circles): white, vehicle; black, isoleucine; gray, valine. Each point represents the mean ± s.e.m for 6–8 individual experiments. * a, *p* < 0.05 Isoleucine *vs*. control; ** b, *p* < 0.05 Valine *vs*. control.

We first asked if increasing the local levels of isoleucine or valine in the MBH of rats modifies circulating glucose levels. To answer this question we performed intrahypothalamic infusions of these BCAAs under basal conditions and measured blood glucose during the course of the infusion. As predicted, both isoleucine and valine individually decreased the plasma levels of glucose compared to vehicle-infused control animals (Figure 2). Thus isoleucine and valine replicated the glucose-lowering action of leucine.

**Figure 3:**
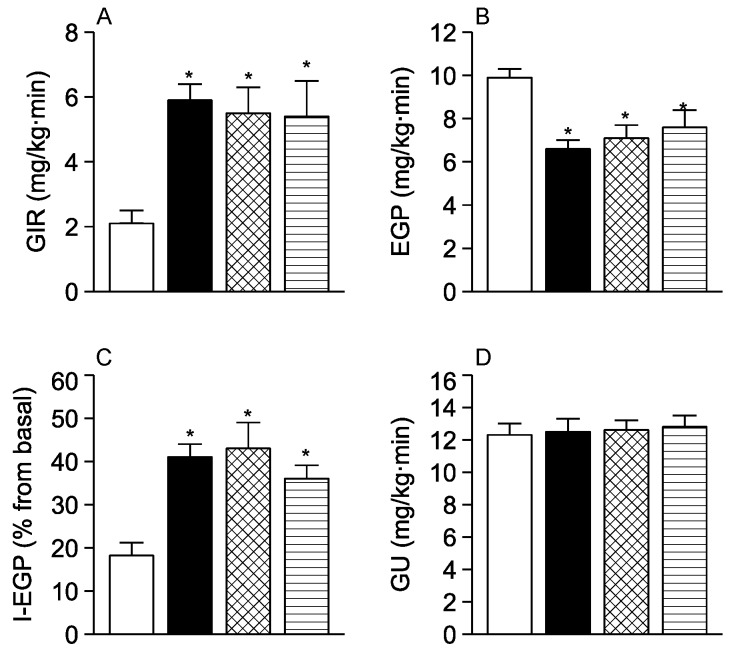
*In vivo* comparison of the central action of isoleucine, valine, and leucine on glucose kinetics during pancreatic insulin clamps. (**A**) Glucose infusion rate (GIR) to maintain euglycemia; (**B**) Endogenous glucose production (EGP); (**C**) Inhibition of EGP by insulin (I-EGP); (**D**) Peripheral glucose utilization (GU). White bars, vehicle; black bars, leucine; check-filled bars, isoleucine; striped bars, valine. Each bar represents the mean ± s.e.m for 6–8 individual experiments. * *p* < 0.05 *vs*. control.

To determine if central isoleucine or valine modulates hepatic glucose metabolism, we performed pancreatic insulin clamps and glucose kinetics analysis in the same animals. During the clamps, when glucose and insulin levels in circulation are kept constant, both isoleucine and valine individually produced a marked increase in the glucose infusion rate (GIR) required to maintain euglycemia compared to vehicle (Figure 3A). Kinetic analysis revealed that the increase of GIR was the result of a marked inhibition of EGP (Figure 3B,C), since peripheral glucose utilization (GU) did not change (Figure 3D). The individual effect of isoleucine and valine on all these kinetic parameters was comparable in magnitude to that reported for leucine [16] at the equimolar dose (12 *p*moles) used here. Taken together these results indicate that isoleucine and valine fully replicated the effect of leucine on liver glucose metabolism. On close examination, the individual potency of isoleucine to inhibit EGP was approximately the same as for leucine but somewhat lower than for valine.

**Figure 4:**
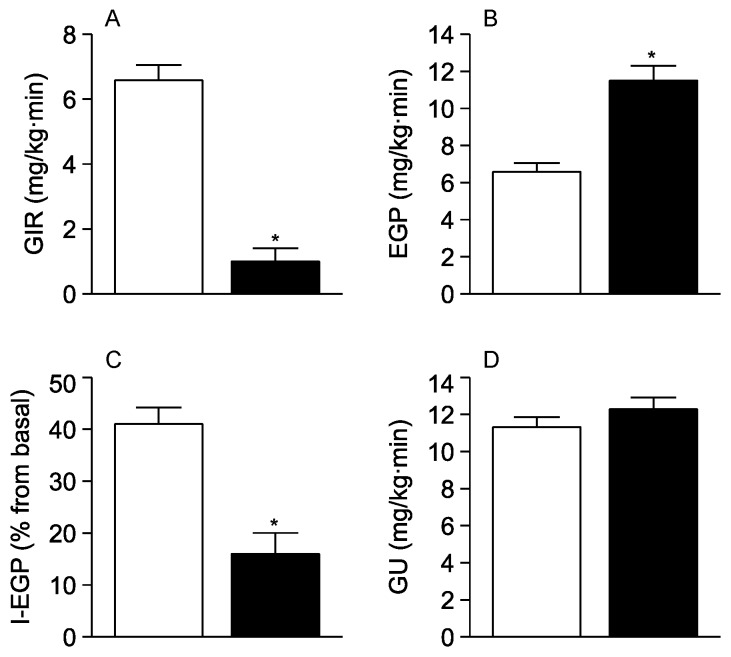
Impact of high-fat diet (HFD) feeding on the central glucoregulatory action of leucine. (**A**) Glucose infusion rate (GIR) to maintain euglycemia; (**B**) Endogenous glucose production (EGP); (**C**) Inhibition of EGP by insulin (I-EGP); (**D**) Peripheral glucose utilization (GU). White bars, regular chow-fed (control) animals; black bars, HFD-fed animals. Each bar represents the mean ± s.e.m for 6 individual experiments. * *p* < 0.05 *vs*. control.

Short-term (3-day) feeding of rodents with a diet enriched in saturated fat (HFD) induces insulin resistance in the absence of changes in body weight compared to regular chow (RC) fed animals [27,28]. To examine the consequences of acutely-induced insulin resistance on the ability of BCAAs to inhibit EGP, we subjected rats to three days of HFD feeding and repeated our measurements during central infusions of leucine. We used leucine because its effects on glucose metabolism have been previously characterized in detail by our group [16]. During pancreatic clamps, acute insulin resistance was manifested by decreased GIR (RC = 2.3 ± 0.6 *vs*. HFD = 0.7 ± 0.2 mg/kg·min; *p* < 0.05) due to an increase of EGP (RC = 9.9 ± 0.4 *vs*. HFD = 11.6 ± 0.6 mg/kg·min; *p* < 0.05) without change in GU (RC = 12.3 ± 0.7 *vs*. HFD = 11.9 ± 0.5 mg/kg·min). Next, as shown in Figure 4A, animals treated with HFD required remarkably less glucose infusion to maintain appropriate plasma glucose levels than animals treated with regular chow (not insulin resistant) when infused centrally with leucine. Analysis of the clamp tracer data revealed that the ability of leucine to reduce EGP was markedly attenuated in these HFD treated animals (Figure 4B,C). Importantly, there was no change in GU (Figure 4D), indicating that all the effect was essentially hepatic. In summary, these results indicate that the short-term consumption of HFD markedly blunts the glucoregulatory action of leucine.

## 4. Discussion

In the current studies, our measurements of whole body glucose kinetics during pancreatic clamps showed that isoleucine and valine each individually modified the main parameters in a very similar way: increased GIR, decreased EGP, and no change in peripheral GU. At an equimolar dose, the magnitude of their effects were comparable to those of leucine. These findings strongly support our hypothesis that all BCAAs are capable of signalling in the MBH through a common upstream metabolic mechanism. Although the goal of our work was to determine whether or not isoleucine and valine had central glucoregulatory activity rather than studying the mechanisms, it would be appropriate to discuss the possibilities. We think that it is the metabolic fate of these BCAAs that allows them to signal in the MBH. In the case of isoleucine, the explanation seems straightforward because this amino acid is directly converted to acetyl-CoA, which can then be used to generate malonyl-CoA. More intriguing, though, is the effect of valine, since this amino acid is not directly metabolized to acetyl-CoA but rather to propionyl-CoA. This metabolite is converted to succinyl-CoA, which then enters the tricarboxylic acid (TCA) cycle, a feature that makes it a glucogenic amino acid. As a glucogenic AA, valine is the second example of its kind, after proline [17], found to modulate glucose metabolism by acting in the hypothalamus. Interestingly, the glucogenic AA histidine was recently shown to inhibit liver glucose production when delivered in the third ventricle of rats, suggesting that it acts in the hypothalamus [29]. However, in contrast with proline, histidine requires binding to histamine receptors rather than its metabolism in the brain to modify liver glucose fluxes. More importantly, we have shown that physiologically relevant elevations of the circulating levels of leucine or proline modulate hepatic glucose metabolism *in vivo* [16,17]. Similarly, it is possible that physiological elevations of isoleucine and valine also regulate circulating glucose levels, but further studies are required to confirm (or rule out) this idea. Several studies in rodents appear to support a role for BCAAs in the improvement of glucose metabolism reported here. For example, dietary leucine supplementation improves glucose metabolism and prevents obesity in various mouse models [30,31]. Furthermore, mice lacking BCAT in the brain display high circulating levels of leucine in association with a lean phenotype and enhanced insulin sensitivity [32]. Interestingly, recent reports of studies in humans have identified a link between elevated levels of circulating BCAAs and the development of insulin resistance or diabetes [33,34]. However, the details of such an association are not clear, and therefore it is currently unknown how the elevated levels of BCAAs are connected to the development of these disorders of glucose metabolism. Interestingly, in one of these studies [33], the administration of BCAA to HFD-fed rodents induced insulin resistance while the administration of BCAA alone did not. These results not only indicate that BCAAs are not directly involved in the development of insulin resistance, but they also coincide with our report in that HFD feeding modifies the metabolic actions of BCAAs in a detrimental fashion. Thus, our current studies showing a blunting of central glucoregulation by leucine after short-term HFD feeding allows us to add amino acids, or leucine at least, to the list of nutrients whose hypothalamic sensing is nutritionally regulated. In this regard, our previous studies showing that molecular disruption of leucine sensing in the MBH precipitates the development of hyperglycemia in rats fed with a high-protein diet [16] further supports the idea that the faltering of central leucine sensing may contribute to disease development.

## 5. Conclusions

The MBH responded to a local increase of isoleucine or valine with an inhibition of EGP, which mainly reflects glucose production by the liver. At an equimolar dose, the magnitude of the individual glucoregulatory effect of either isoleucine or valine was similar to that of leucine. Importantly, insulin resistance induced by short-term high fat feeding markedly attenuated the central glucoregulatory effect of leucine. In summary, we conclude that not only leucine but also circulating isoleucine and valine may participate in the acute postprandial regulation of glycemia. Furthermore, consumption of diets rich in saturated fat incapacitates this centrally-mediated glucoregulation.

## References

[1] Eaton S.B., Konner M. (1985). Paleolithic nutrition. A consideration of its nature and current implications. N. Engl. J. Med..

[2] Hill J.O., Peters J.C. (1998). Environmental contributions to the obesity epidemic. Science.

[3] Ukropec J., Sebokova E., Klimes I. (2001). Nutrient sensing, leptin and insulin action. Arch. Physiol. Biochem..

[4] Obici S., Rossetti L. (2003). Minireview: Nutrient sensing and the regulation of insulin action and energy balance. Endocrinology.

[5] Lindsley J.E., Rutter J. (2004). Nutrient sensing and metabolic decisions. Comp. Biochem. Physiol. B Biochem. Mol. Biol..

[6] Schwartz G.J. (2004). Biology of eating behavior in obesity. Obes. Res..

[7] Rossetti L., Rothman D.L., DeFronzo R.A., Shulman G.I. (1989). Effect of dietary protein on *in vivo* insulin action and liver glycogen repletion. Am. J. Physiol..

[8] Patti M.E., Brambilla E., Luzi L., Landaker E.J., Kahn C.R. (1998). Bidirectional modulation of insulin action by amino acids. J. Clin. Investig..

[9] Gannon M.C., Nuttall F.Q., Saeed A., Jordan K., Hoover H. (2003). An increase in dietary protein improves the blood glucose response in persons with type 2 diabetes. Am. J. Clin. Nutr..

[10] Nuttall F.Q., Gannon M.C., Saeed A., Jordan K., Hoover H. (2003). The metabolic response of subjects with type 2 diabetes to a high-protein, weight-maintenance diet. J. Clin. Endocrinol. Metab..

[11] Tremblay F., Krebs M., Dombrowski L., Brehm A., Bernroider E., Roth E., Nowotny P., Waldhausl W., Marette A., Roden M. (2005). Overactivation of S6 kinase 1 as a cause of human insulin resistance during increased amino acid availability. Diabetes.

[12] Promintzer M., Krebs M. (2006). Effects of dietary protein on glucose homeostasis. Curr. Opin. Clin. Nutr. Metab. Care.

[13] Tremblay F., Lavigne C., Jacques H., Marette A. (2007). Role of dietary proteins and amino acids in the pathogenesis of insulin resistance. Annu. Rev. Nutr..

[14] Cota D., Proulx K., Smith K.A., Kozma S.C., Thomas G., Woods S.C., Seeley R.J. (2006). Hypothalamic mTOR signaling regulates food intake. Science.

[15] Blouet C., Jo Y.H., Li X., Schwartz G.J. (2009). Mediobasal hypothalamic leucine sensing regulates food intake through activation of a hypothalamus-brainstem circuit. J. Neurosci..

[16] Su Y., Lam T.K., He W., Pocai A., Bryan J., Aguilar-Bryan L., Gutierrez-Juarez R. (2012). Hypothalamic leucine metabolism regulates liver glucose production. Diabetes.

[17] Arrieta-Cruz I., Su Y., Knight C.M., Lam T.K.T., Gutiérrez-Juárez R. (2013). Evidence for a role of proline and hypothalamic astrocytes in the regulation of glucose metabolism in rats. Diabetes.

[18] Suryawan A., Hawes J.W., Harris R.A., Shimomura Y., Jenkins A.E., Hutson S.M. (1998). A molecular model of human branched-chain amino acid metabolism. Am. J. Clin. Nutr..

[19] Brosnan J.T., Brosnan M.E. (2006). Branched-chain amino acids: Enzyme and substrate regulation. J. Nutr..

[20] Morgan K., Obici S., Rossetti L. (2004). Hypothalamic responses to long-chain fatty acids are nutritionally regulated. J. Biol. Chem..

[21] Lam T.K., Gutierrez-Juarez R., Pocai A., Rossetti L. (2005). Regulation of blood glucose by hypothalamic pyruvate metabolism. Science.

[22] Pocai A., Lam T.K., Obici S., Gutierrez-Juarez R., Muse E.D., Arduini A., Rossetti L. (2006). Restoration of hypothalamic lipid sensing normalizes energy and glucose homeostasis in overfed rats. J. Clin. Investig..

[23] Lam T.K., Gutierrez-Juarez R., Pocai A., Bhanot S., Tso P., Schwartz G.J., Rossetti L. (2007). Brain glucose metabolism controls the hepatic secretion of triglyceride-rich lipoproteins. Nat. Med..

[24] Paxinos G., Watson C. (2007). The Rat Brain in Stereotaxic Coordinates.

[25] Gutierrez-Juarez R., Obici S., Rossetti L. (2004). Melanocortin-independent effects of leptin on hepatic glucose fluxes. J. Biol. Chem..

[26] Liu L., Karkanias G.B., Morales J.C., Hawkins M., Barzilai N., Wang J., Rossetti L. (1998). Intracerebroventricular leptin regulates hepatic but not peripheral glucose fluxes. J. Biol. Chem..

[27] Kraegen E.W., Clark P.W., Jenkins A.B., Daley E.A., Chisholm D.J., Storlien L.H. (1991). Development of muscle insulin resistance after liver insulin resistance in high-fat-fed rats. Diabetes.

[28] Wang J., Obici S., Morgan K., Barzilai N., Feng Z., Rossetti L. (2001). Overfeeding rapidly induces leptin and insulin resistance. Diabetes.

[29] Kimura K., Nakamura Y., Inaba Y., Matsumoto M., Kido Y., Asahara S.I., Matsuda T., Watanabe H., Maeda A., Inagaki F. (2013). Histidine augments the suppression of hepatic glucose production by central insulin action. Diabetes.

[30] Zhang Y., Guo K., LeBlanc R.E., Loh D., Schwartz G.J., Yu Y.H. (2007). Increasing dietary leucine intake reduces diet-induced obesity and improves glucose and cholesterol metabolism in mice via multimechanisms. Diabetes.

[31] Guo K., Yu Y.H., Hou J., Zhang Y. (2010). Chronic leucine supplementation improves glycemic control in etiologically distinct mouse models of obesity and diabetes mellitus. Nutr. Metab. (Lond.).

[32] She P., Reid T.M., Bronson S.K., Vary T.C., Hajnal A., Lynch C.J., Hutson S.M. (2007). Disruption of BCATm in mice leads to increased energy expenditure associated with the activation of a futile protein turnover cycle. Cell Metab..

[33] Newgard C.B., An J., Bain J.R., Muehlbauer M.J., Stevens R.D., Lien L.F., Haqq A.M., Shah S.H., Arlotto M., Slentz C.A. (2009). A branched-chain amino acid-related metabolic signature that differentiates obese and lean humans and contributes to insulin resistance. Cell Metab..

[34] Wang T.J., Larson M.G., Vasan R.S., Cheng S., Rhee E.P., McCabe E., Lewis G.D., Fox C.S., Jacques P.F., Fernandez C. (2011). Metabolite profiles and the risk of developing diabetes. Nat. Med..

